# Investigation of Water‐Soluble Binders for LiNi_0.5_Mn_1.5_O_4_‐Based Full Cells

**DOI:** 10.1002/open.202200065

**Published:** 2022-06-14

**Authors:** Girish D. Salian, Jonathan Højberg, Christian Fink Elkjær, Yonas Tesfamhret, Guiomar Hernández, Matthew J. Lacey, Reza Younesi

**Affiliations:** ^1^ Department of Chemistry-Ångström Laboratory Uppsala University Box 538 75121 Uppsala Sweden; ^2^ Haldor Topsøe A/S Haldor Topsøes Allé 1 2800 Kgs Lyngby Denmark; ^3^ Scania CV AB 151 87 Södertälje Sweden

**Keywords:** aqueous binders, lithium nickel manganese oxide, lithium titanate, Li-ion

## Abstract

Two water‐soluble binders of carboxymethyl cellulose (CMC) and sodium alginate (SA) have been studied in comparison with *N*‐methylpyrrolidone‐soluble poly(vinylidene difluoride–*co*‐hexafluoropropylene) (PVdF‐HFP) to understand their effect on the electrochemical performance of a high‐voltage lithium nickel manganese oxide (LNMO) cathode. The electrochemical performance has been investigated in full cells using a Li_4_Ti_5_O_12_ (LTO) anode. At room temperature, LNMO cathodes prepared with aqueous binders provided a similar electrochemical performance as those prepared with PVdF‐HFP. However, at 55 °C, the full cells containing LNMO with the aqueous binders showed higher cycling stability. The results are supported by intermittent current interruption resistance measurements, wherein the electrodes with SA showed lower resistance. The surface layer formed on the electrodes after cycling has been characterized by X‐ray photoelectron spectroscopy. The amount of transition metal dissolutions was comparable for all three cells. However, the amount of hydrogen fluoride (HF) content in the electrolyte cycled at 55 °C is lower in the cell with the SA binder. These results suggest that use of water‐soluble binders could provide a practical and more sustainable alternative to PVdF‐based binders for the fabrication of LNMO electrodes.

## Graphical abstract

**Water‐based binders for LiNi_0.5_Mn_1.5_O_4_ cathodes** have been studied in full cells. LNMO cathodes containing sodium alginate binder give the lowest internal resistance, especially if operated at 55 °C. Reduced side reactions due to the protective nature of sodium alginate for LNMO at high potentials could be a reason for the better cyclability and lower internal resistance that have measured.

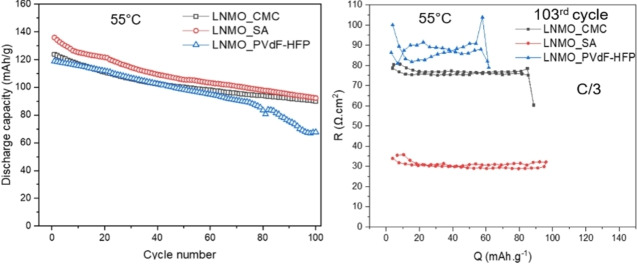

## Introduction

Spinel LiNi_0.5_Mn_1.5_O_4_ (LNMO) is one of the promising cathode materials for high energy density lithium‐ion batteries (LIBs) due to its advantages like high operating voltage (4.7 V vs. Li/Li^+^), high reversible capacity (theoretical capacity is 147 mAh g^−1^), good thermal stability, high power, low cost and environment friendliness.[^1^, ^2^, ^3^] LNMO, being a cobalt‐free cathode, becomes more prominent in the current scenario where there is a continuous rise of cobalt prices and shortages in supply chain.^[2]^ Due to its energy density and high‐rate capabilities, there is much demand in the commercialization of LNMO for lithium‐ion batteries in electric vehicle and high‐power applications. However, the operation of LNMO‐based cells at high voltage results in parasitic reactions due to electrolyte decomposition and transition metal (TM) dissolution, especially at elevated temperatures, hence negatively affecting the cell cyclability.[^4^, ^5^, ^6^] Although an electrochemically inactive component, the binder plays a crucial role in the long‐term cycle performance of the electrodes by allowing a homogeneous distribution of the conducting additive and active material. This ensures a good electronic contact upon cycling for electron transfer and facilitates the formation of a stable interface with the electrolyte.[^7^, ^8^] The presently used polyvinylidene difluoride (PVdF) binder requires *N*‐methylpyrrolidone (NMP), an organic solvent which is environmentally harmful, for the electrode preparation. Hence, there is a strong interest to use water‐soluble binders which are low‐cost, safe and environmentally friendly.

The most commonly used water‐soluble binder is carboxymethyl cellulose (CMC) for both cathodes and anodes in LIBs.[^9^, ^10^, ^11^, ^12^, ^13^] CMC and its variants have also been used in LNMO cathodes and have shown relatively good discharge capacities and retention compared to PVdF binder. Francesca et al.^[14]^ presented LNMO‐Graphite full cells wherein both electrodes were processed using CMC binders, with a capacity retention of 83 % at 1 C rate for 400 cycles. They attributed the cycling stability to the formation of a thin passivation layer on LNMO which helped in mitigating the undesired reactions at high potentials. Kuenzel et al.^[15]^ showed that by crosslinking natural guar gum to CMC assisted by citric acid, the resultant aqueous binder, when used with LNMO and graphite full cells, could perform at 80 % capacity retention after 1000 cycles at 1 C. Chou et al.^[16]^ showed that the protective film formed on LNMO processed with CMC binder effectively reduced the contact between LNMO and the electrolyte, thereby suppressing the transition metal dissolution.

Efficient drying of the electrodes processed in water is also a very important step. Ngyuen et al. suggested that the poor performance of the LNMO electrodes processed with CMC binder was due to trace amounts of water which reacted with the EC:DEC+1 m LiPF_6_ electrolyte, producing HF.^[17]^ As a consequence, efficient drying is a particularly important factor in the industry where electrodes are coated and dried in a dry room where there could be still some contribution from the moisture. However, at the lab level, efficient drying is often done in a vacuum oven before assembly of electrodes into cells.

Another efficient and recently investigated aqueous binder is sodium alginate (SA), which is a biopolymer extracted from sea weeds. The carboxylic groups present in this polymer facilitate the formation of hydrogen bonds between the electrode and the binder.^[18]^ SA has mainly been used in anodes; however, it has found application in the fabrication of LiMn_2_O_4_ (LMO) electrodes, wherein the protective film formed on LMO could act as Mn^2+^ scavenger.^[18]^ Bigoni et al.^[19]^ used SA for making LNMO electrodes and used them in half cells. They showed 86 % capacity retention after 200 cycles at 1 C rate. However, to date, there are no reports on the use of SA binders in LNMO based full‐cells.

All the above‐detailed positive reports on incorporating water‐soluble binders highlight the importance of the cathode electrolyte interface (CEI) which forms on LNMO cathodes. This is crucial in mitigating the side reactions and TM dissolution from LNMO. We present results on LNMO‐LTO full cells wherein the LNMO electrodes were processed using water‐based CMC and SA binders. LTO as the counter electrode, instead of commonly used Li metal, has been chosen to avoid the detrimental effect of Li‐metal and its side products on high‐voltage LNMO electrodes.^[20]^ As a comparison, the LNMO electrodes were also prepared using PVdF‐HFP binder dissolved in NMP solvent. Considering the high‐voltage cycling of LNMO‐based cells, apart from the electrolyte oxidation, two important factors deter the cycling stability, namely TM dissolution and hydrogen fluoride (HF) formation at high potentials.^[5]^ TM dissolution is accelerated at high voltages to form Mn^2+^ which is soluble in the electrolyte and HF formation could trigger this dissolution even more. Thus, when it comes to use of water‐soluble or ‐based binders, formation of HF is a serious deterrent, if there is any remnant water present in the electrode. Therefore, we here also investigate the presence of HF formation and its effect on the TM dissolution especially at elevated temperatures.

## Results and Discussion

**Figure 1:**
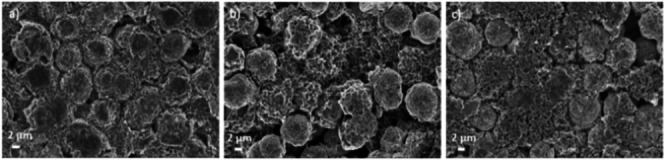
SEM images of pristine LNMO electrodes with different binders of a) CMC; b) SA; c) PVdF‐HFP.

Figure 1 shows the SEM images of the surface view of the LNMO composite electrodes prepared with the three different binders, that is, CMC, SA, and PVdF‐HFP. The LNMO particle size as seen in the image is around ≈5 μm. The effect of calendaring is also observed on the top surface of the LNMO particles. The network of small particles, which also contains the carbon black, is shown to spread throughout the LNMO particle. The images show that there is no marked difference in the morphology considering the three electrodes.

**Figure 2:**
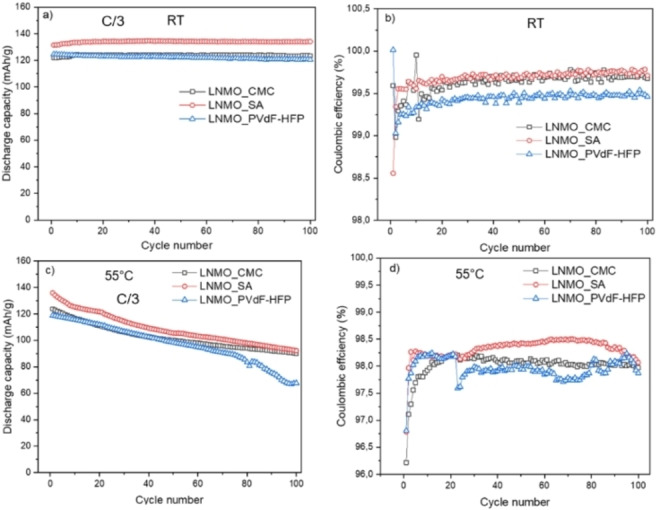
a) Discharge capacities and Coulombic efficiencies obtained from galvanostatic cycling of LNMO_CMC, LNMO_SA and LNMO_PVdF‐HFP full‐cells using LTO as the anode. The cells were cycled at C/3 rate at RT (a and b); and at 55 °C (c and d).

Figure 2 shows the cycling performances of LNMO‐LTO full cells cycled at C/3 rate at two different temperatures of RT and 55 °C. The cells with LNMO electrodes with different binders are marked as LNMO_CMC, LNMO_SA and LNMO_PVdF‐HFP. Prior to the cycling at C/3 for 100 cycles, each cell was subjected to a formation cycle of 3 cycles at C/10 rate (see Figure S1). The cycles at C/3 rate from 4^th^ cycle to 103^rd^ cycle are discussed here.

At RT, all three cells with CMC, SA and PVdF‐HFP binders show stable capacities up to the 103^rd^ cycle (Figure 2a). For the cells with the water‐soluble binders, there is a slight increase in capacity during the initial cycles which could be attributed to the eventual wetting of the electrode by the electrolyte. The Coulombic efficiencies (CE) of the cells with SA and CMC were >99.5 % marking an excellent reversibility in comparison to PVdF‐HFP (≈99.3 %; Figure 2b). At 55 °C, as seen in Figure 2c, the 4^th^ cycle capacity for LNMO_SA is 135 mA h g^−1^ compared to the other two cells. At the 103^rd^ cycle, the cells with SA and CMC retain more capacity compared to the cell with PVdF‐HFP. Thus, the cells with the water‐soluble binders showed higher capacity retentions. The CE for the cells with SA and CMC binders were approximately ≥98 %, while the cell with PVdF‐HFP performed with CE varied between 97–98 %. Interestingly, for the cell with LNMO_SA, the CE increases to up to 98.5 %, but after 80 cycles it eventually starts decreasing to 98 %.

**Figure 3:**
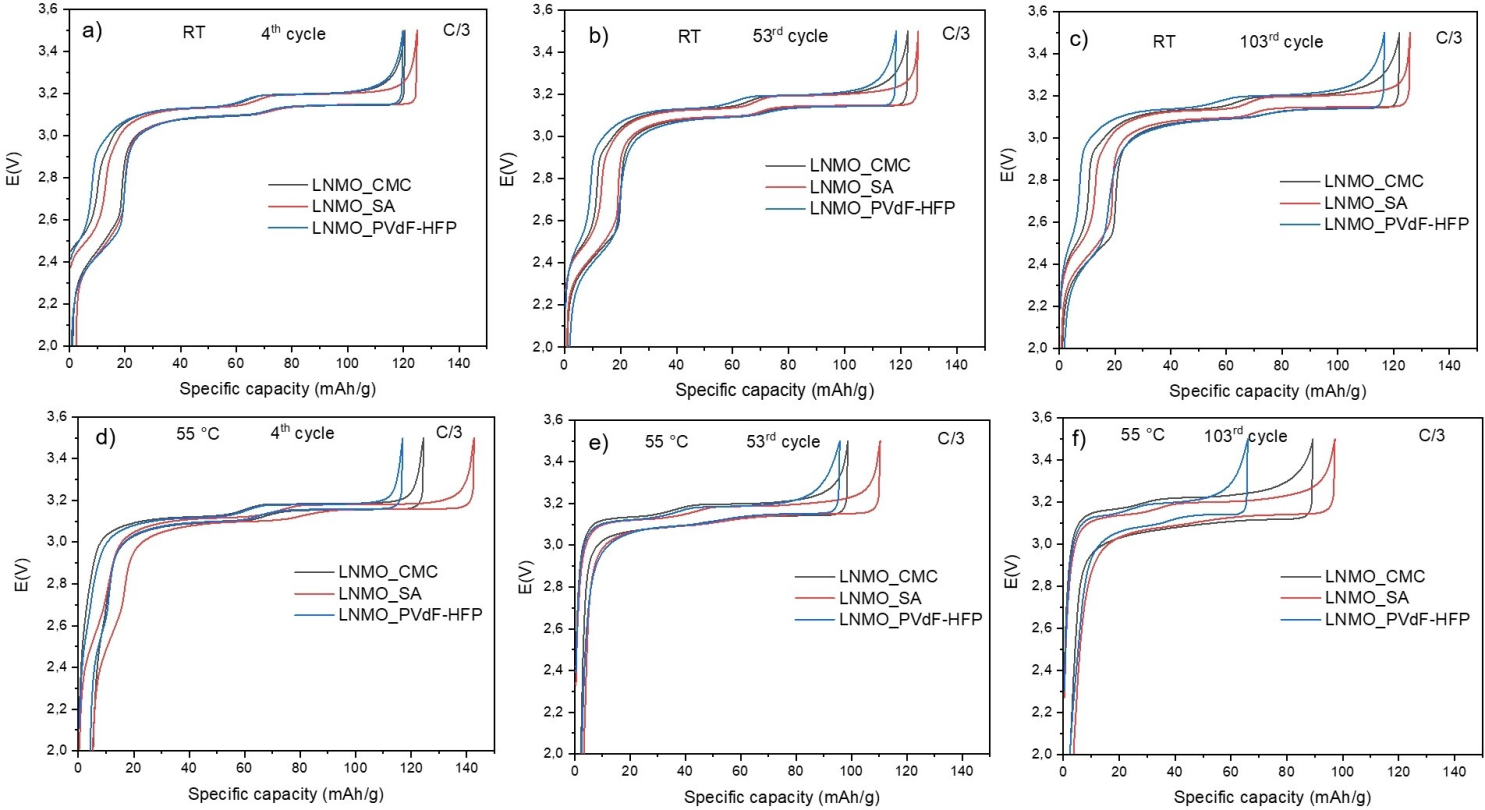
Galvanostatic voltage profiles of LNMO_CMC, LNMO_SA and LNMO_PVdF‐HFP full‐cells at C/3 rate for different cycles a), b), c) at RT and d), e) and f) at 55 °C.

Figure 3 shows the galvanostatic charge/discharge profiles of the selected cycles of the full‐cells at C/3 rate. The profiles of the cells cycled at RT (Figures 3a–c) indicate that the overpotential for the cell with LNMO_SA remains the same whereas for the cells LNMO_CMC and LNMO_PVdF‐HFP it increases relatively at the 53^rd^ and the 103^rd^ cycles. Similarly, for the full cells cycled at 55 °C (Figures 3d –f), the cell with LNMO_SA shows a lower overpotential increase than the other two cells. The overpotential of the 4^th^ cycles of all the three cells is lower at 55 °C compared to that at RT due to improved kinetics at higher temperature.^[3]^ The voltage profiles both at RT and 55 °C could suggest the formation of more resistive and thicker surface layers for the cells with LNMO_PVdF‐HFP and LNMO_CMC, which is more evident at 55 °C. The interfacial resistance for LNMO_SA can be considered to be more stable compared to the cells with LNMO_CMC and LNMO_PVdF‐HFP.

**Figure 4:**
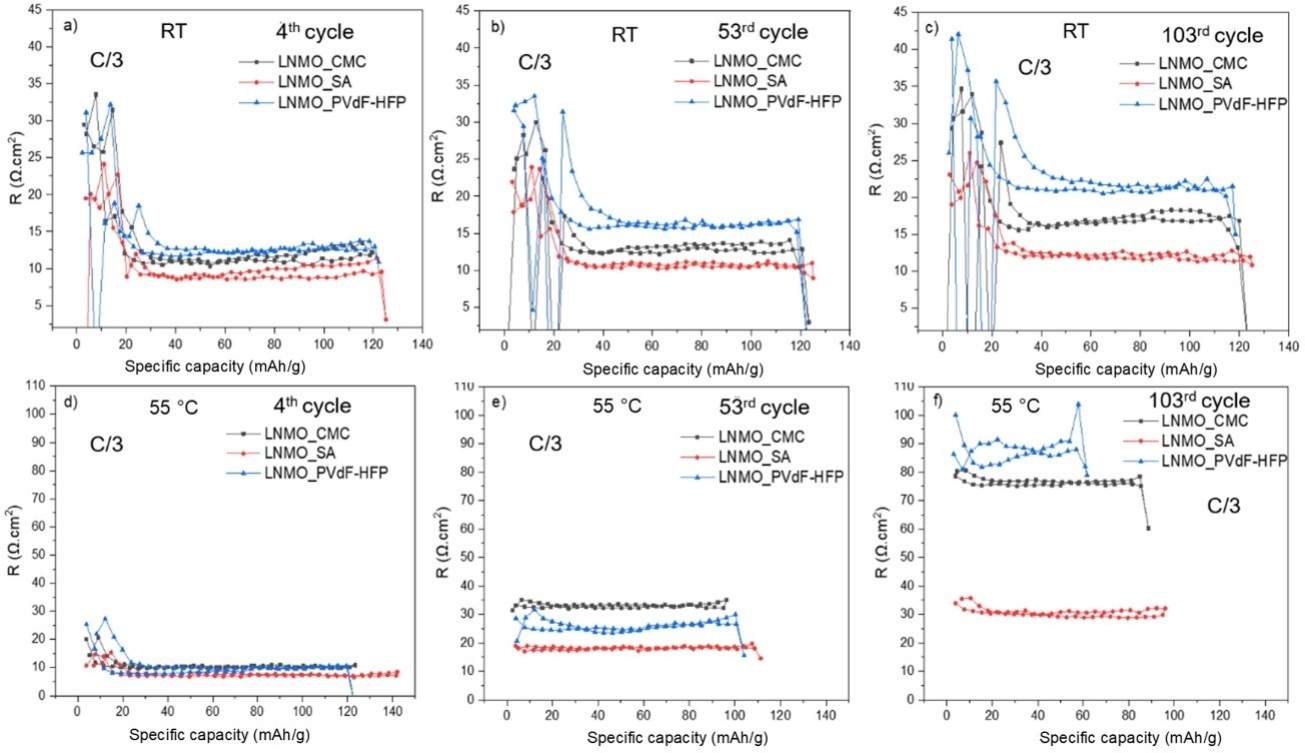
Resistance profiles of LNMO‐LTO full cells at different cycles with the three different binders studied here. The ICI tests were performed at room temperature (a, b, c), and at 55 °C (d, e, f).

To further investigate the internal resistances, galvanostatic charge‐discharge cycling were combined with intermittent current interruption (ICI) experiments. Figure 4 displays the cell resistance plotted versus the capacity for all the cells. At RT, the resistance in the cell containing LNMO_PVdF‐HFP increased from the 4^th^ cycle up to the 103^rd^ cycle in comparison to its counterpart cells containing the water‐soluble binders. The cell with LNMO_SA showed the lowest cell resistance even at the 103^rd^ cycle. At 55 °C, the differences on the cell resistance are more noticeable. After 103 cycles at 55 °C, the cells with LNMO_CMC and LNMO_PVdF‐HFP had much larger resistance compared to that at RT, while the cell with LNMO_SA showed a slight increase in resistance to about 30 Ω cm^2^. At the 53^rd^ cycle, the cell with LNMO_CMC showed a higher resistance than the other two cells but, at the 103^rd^ cycle, the cell resistance was higher for the cell with LNMO_PVdF‐HFP. The ICI studies well corroborates with the overpotential increase shown in Figure 3, especially for the tests performed at 55 °C. The ICI measurements for the formation cycles of all the three cells at RT and 55 °C are shown in Figure S2.

The cells tested in the resistance measurements are in two‐electrode configuration. Hence, apart from the resistance from LNMO, the LTO anodes may also contribute to the total cell resistance. It has previously been shown that surface species can be formed on the surface of LTO, although the potential of LTO is within the stability window of organic carbonate‐based electrolytes.^[21]^ For LNMO‐LTO cells, Dedryvere et al.^[22]^ and Li et al.^[23]^ have shown that surface species are first formed at the positive electrode before being adsorbed at the LTO surface either by diffusion or by migration of organic cationic species. Here, our results also showed formation of surface species on LTO electrodes (see Figure S3); however, the XPS spectra are quite similar for all LTO electrodes. Therefore, the choice of binder in LNMO showed no major impact on the surface composition of LTO electrodes (note that all LTO electrode were prepared with CMC‐SBR binder. Therefore, the choice of binder in LNMO suggests no major impact on the surface composition of LTO electrodes.

Another aspect to be noted is that, although the resistance at 55 °C was much lower for the LNMO_SA compared to that for LNMO_CMC and LNMO_PVdF‐HFP, the capacity fading is almost similar for all the cells at 55 °C. Therefore, the capacity fading is likely due to the loss of cyclable lithium consumed via the side reactions at 55 °C.[^3^, ^24^] It is worth mentioning that the electrode integrity after cycling remained intact as no cracking or delamination was observed in SEM analysis of cycled electrodes at RT and at 55 °C (see Figure S4).

**Figure 5:**
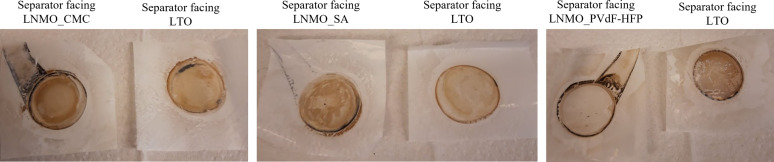
Celgard separator photographs taken after disassembling the cells showing the solid products deposited on it at 55 °C.

The color change in separators was observed for all the cells cycled at 55 °C (see Figure 5), which indicates that deposition of solid side products on the separators. Also, the concentration of HF (see Table 1) in the electrolytes retrieved from all the cycled cells at 55 °C was much higher than that in the pristine electrolyte (note that the HF measurements were carried out in cells with higher volume of the electrolyte, 500 μL). The HF concentration was, however, found to be slightly smaller in the cell with LNMO_SA when compared to the other cells.

**Table 1:** HF concentrations (in ppm) present in the electrolyte retrieved from LNMO‐LTO full cells after 100 cycles at 55 °C.

	HF concentration (in ppm)
LP40 (Reference at RT)	98
LNMO_SA at 55 °C	2587
LNMO_CMC at 55 °C	3116
LNMO_PVdF‐HFP at 55 °C	3286

NMR measurements in DMSO‐d_6_ were carried out on the same retrieved electrolyte at 55 °C, to understand the soluble decomposition products present in the cycled electrolyte (see Figure S5). The aim was to observe if there would be any difference in the constituents of the degradation products in the retrieved electrolyte. For the ^1^H NMR results (Figures S5 a and b), the peak at 4.2 ppm can be assigned to the ring‐opened ethylene carbonate (−CH_2_OC(=O)O−) which can lead to ether units (−OCH_2_−) after decarboxylation as confirmed by the peak at 3.5 ppm.[^25^, ^26^, ^27^] Small differences in chemical shift with other reported values might be due to different ratios of carbonate to ether units, different chain lengths and/or variation in terminal groups.^[25]^ Furthermore, the triplet around 1 ppm together with the doublet at 3.4 ppm could be assigned to the degradation of diethyl carbonate forming ethanol or diethyl ether, also releasing CO_2_.[^28^, ^29^, ^30^] The electrolytes from all three cells contain HF, confirmed by the appearance of the peaks at −167.7 and −190 ppm corresponding to HF in various coordination environments and complexations,^[31]^ and a peak at −148 ppm in the ^19^F NMR spectra (Figures S5 c and d), corresponding to BF_4_^−^ as a result of the reaction between HF and the glass from NMR tube.[^32^, ^33^] One notable difference between these spectra is that the electrolyte from the cells with LNMO_CMC and LNMO_PVdF‐HFP showed a doublet at −69.7 ppm which could confirm residues of fluorophosphates.^[32]^ However, the cell with LNMO_CMC does not show any peak in the ^31^P NMR spectrum while the cell with LNMO_PVdF‐HFP shows one peak at 0 ppm typically assigned to phosphate molecules (PO_4_^3−^).^[34]^ In contrast, the cell with LNMO_SA does neither show any phosphorous nor fluorinated species except for HF. It is important to highlight that the samples were exposed to ambient air which could contribute to further LiPF_6_ hydrolysis in addition to what had happened inside the cell. Furthermore, the samples contained some precipitates which are not analyzed with solution NMR spectroscopy but have been previously reported to be a three‐dimensional fluorinated and phosphorous polymer network formed from the hydrolysis of LiPF_6_ and organic solvents at elevated temperatures.^[34]^ Another possible explanation why we do not see fluorinated and/or phosphorous species in the NMR spectra could be that the concentration is below the detection limit of the NMR spectrometer. Overall, while the ^1^H spectra show similar degradation products in the electrolytes from the three cells, ^19^F and ^31^P NMR spectra show different degradation mechanisms depending on the binder used for LNMO.

The amount of dissolved Mn and Ni in the electrolyte (see Table 2) of cells cycled at 55 °C was however similar for all the cells (note that the tests were performed at 55 °C to observe the TM dissolution at a higher temperature using a different cycling protocol where the cells were cycled at C/10 for 5 cycles and left in charged state in the 6^th^ cycle for 48 h). Since the ICP‐OES results reveal that the amount of dissolved transition metals in the cell with LNMO_PVdF‐HFP is slightly higher, it can be concluded that the aqueous binders are comparable to the performance of the PVdF‐HFP binder. This suggests that there could be contribution to the internal resistance or capacity fade from the TM dissolution, but it is not considerable. Kim et al.^[24]^ reported that the contribution to the capacity fading due to TM dissolution in a LNMO‐Graphite full cell was less than 5 %.

**Table 2:** ICP results for the electrolyte retrieved from the LNMO‐LTO full‐cells cycled at 55 °C.

	Amount of Mn [ppm or mg L^−1^]	Amount of Ni [ppm or mg L^−1^]
Electrolyte from LNMO_SA	0.43	0.11
Electrolyte from LNMO_CMC	0.46	0.10
Electrolyte from LNMO_PVdF‐HFP	0.53	0.17

All the aforementioned results reveal the presence of side reactions leading to capacity loss. However, the lower internal resistance in the cell with LNMO_SA suggests that some of side products are adsorbed by the SA binder, thus leading to lower resistance on the separator in the LNMO_SA cell. This is in line with the study by Ryou et al.,^[18]^ where the beneficial influence of sodium alginate binder on LiMn_2_O_4_ was shown. They proposed an egg‐box model wherein the functional groups of the alginate binder can capture the manganese and prevent them from going into the electrolyte.^[18]^ It is likely that the same mechanism leads to capture side products by SA here. Also, the lower resistance for LNMO_SA could suggest that SA offers a ‘protective’ effect on the LNMO particles, reducing the extent of electrolyte oxidation and thus leading to lower HF production and TM dissolution. This protective effect is reflected in the CE of LNMO_SA cycled at 55 °C, that is, it shows higher CE compared to other cells. Hence the loss of cyclable lithium is lower, indicating lower side reactions and hence lower HF formation and TM dissolution.

To understand whether the parasitic reactions led to the formation of any surface layer, XPS analysis was performed on the pristine and cycled LNMO and LTO electrodes. Figure S6a shows the survey spectra of the pristine LNMO electrodes without exposure to the electrolyte. The XPS survey spectra for the LNMO_PVdF‐HFP show the characteristic peaks of F 1s around 688 eV corresponding to the binder. The Na 1s peak observed in the spectra of LNMO_CMC and LNMO_SA originates from the sodium salt of the CMC and SA binders. The peaks that correspond to C 1s and O 1s in all the three electrodes arise from the carbon additives and the binder themselves. The Mn 2p and Ni 2p peaks are characteristic of LNMO.

Figures S6 b and c show the survey spectra of the cycled LNMO electrodes (after 103 cycles) both at RT and 55 °C. Since the Na 1s peak for the LNMO_CMC and LNMO_SA is not observed in the cycled LNMO electrodes, one can conclude that a surface layer formed. The emergence of the F 1s peak for the electrodes LNMO_CMC and LNMO_SA at both temperatures points to a fluorine‐rich surface layer. The contribution of F 1s comes from the interaction with the electrolyte containing LiPF_6_. Along with the binder peak, the F 1s peak observed for LNMO_PVdF‐HFP also contains the peak arising from the surface layer. Additionally, the C 1s and O 1s peaks for the cycled electrodes are attributed to the evolution of organic/inorganic species due to the oxidation of the electrolyte. The Mn 2p and Ni 2p peaks corresponding to LNMO are still observed after 100 cycles, suggesting that the surface layer is thinner than 10 nm (rough estimation of probing depth of XPS with Al‐K_α_ source).

**Figure 6:**
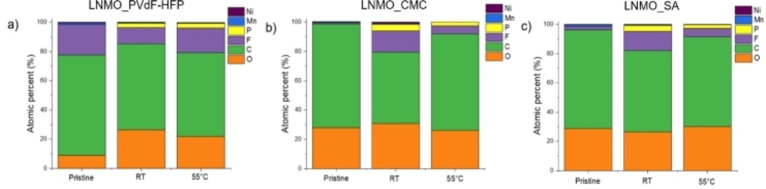
Atomic percentages obtained from the survey spectra of the pristine and the cycled LNMO electrodes a) LNMO_PVdF‐HFP, b) LNMO_CMC; c) LNMO_SA.

The influence of the formed species on the surface layer was analyzed with respect to the atomic percentages of the different elements from the survey spectra of the pristine and the cycled electrodes as presented in Figure 6. For LNMO_CMC and LNMO_SA, it is clear that fluorine detected in the cycled samples originated from decomposition of the electrolyte salt, LiPF_6_. The amount of F, however, is lower on the surface of samples cycled at 55 °C compared to those cycled at RT. This suggests that the decomposed species are dissolved in the electrolyte as the solubility of solid side products in the electrolyte increases with the temperature. Alternatively, it could have led to the formation of solid side products on the separator. This is in line with results depicted in Figure 5 indicating that more side products formed on the separator at 55 °C. For all the cycled electrodes, there is also the emergence of phosphorus‐containing species coming from the salt degradation. This could also explain the lack or low amount of salt degradation products (phosphorous and fluorine species) observed from NMR spectroscopy.

**Figure 7:**
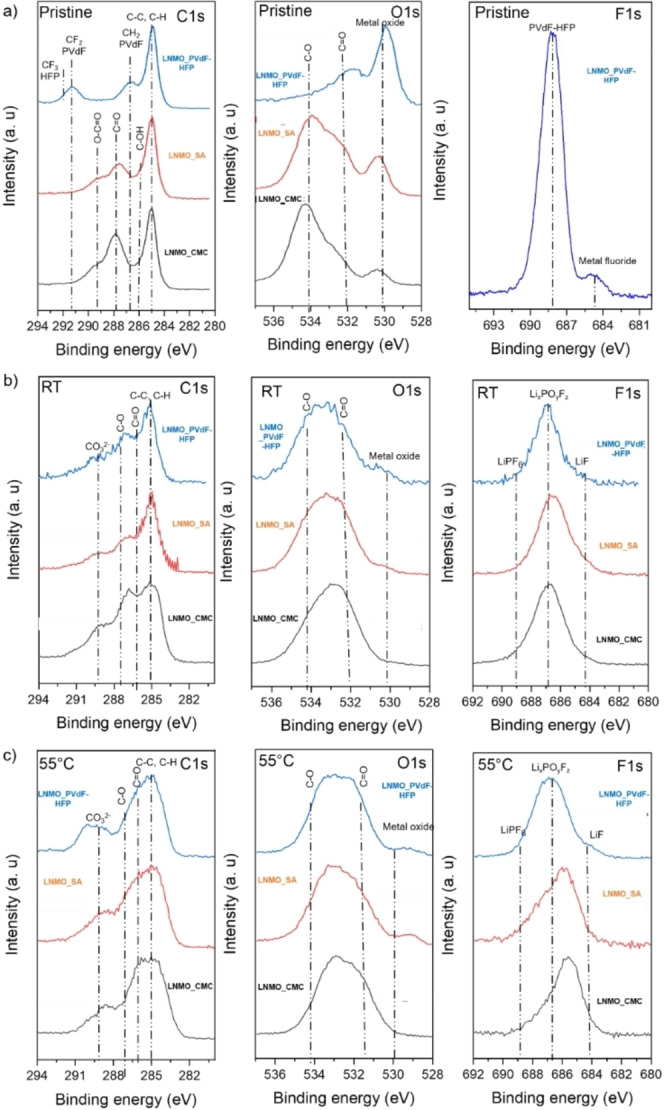
C1s, O1s and F1s XPS spectra for the a) pristine and cycled LNMO electrodes at b) RT and c) 55 °C.

Figure 7 shows the individual spectra of C 1s, O 1s and F 1s for the pristine and the cycled (after 103 cycles) LNMO electrodes in full cells. For all the pristine LNMO electrodes, a large contribution appears from carbon black and hydrocarbon which is assigned to the peak at 285.0 eV (all spectra were calibrated with respect to the C−C peak). The pristine LNMO_PVdF‐HFP electrode shows the characteristic peaks related to the binder (−CF_2_, −CF_3_) in the C 1s spectra. In the O 1s spectra, along with the metal oxide peak at 530.1 eV, we could also assign C=O‐ and C−O‐related adsorbed species. The F 1s spectrum shows one large peak around 688 eV, corresponding to the PVdF–HFP binder. Also, in the F 1s spectrum, a small peak at 685 eV is observed which could be assigned to a metal fluoride. For the pristine electrodes of LNMO_CMC and LNMO_SA, along with the C−C peak, the binder peaks for C=O, O−C=O and C−OH at 287.8 eV, 289.3 eV and 286.3 eV are assigned. In the O 1s spectra for both pristine electrodes with aqueous binders, the C−O and C=O peaks are very prominent and have been assigned to their respective binders.

For the electrodes cycled at RT (after 103 cycles), the surface layers as observed in the C 1s spectra of all three electrodes, predominantly consist of species with C=O, C−O, and carbonate peaks (Li_2_CO_3_). The binder peaks get inhibited in this case due to the formation of these surface layers. The C 1s spectrum of LNMO_CMC shows higher peak intensities around the peaks for C=O and C−O, which could suggest a higher amount of these species being formed. In the O 1s spectra for all three electrodes, the metal oxide peak intensity subsides when compared to the pristine ones suggesting the formation of surface layers. The F 1s spectra consist of peaks corresponding to Li_x_PO_y_F_z_, LiPF_6_ (which could come from the remnant salts from the electrolyte) and LiF or metal fluorides (NiF_2_ or MnF_2_). For the electrodes cycled at 55 °C, the concentration of the carbon species increases, overlapping with the C−C peak from the carbon black. This is seen in the C 1s spectra of all three electrodes. The same peaks as for the electrodes cycled at RT could be assigned here for all the C 1s, O 1s and F 1s spectra. The only observable difference could relate to a higher concentration of the species formed at elevated temperatures when compared to the RT case.

The nature of the surface layers formed on the three electrodes after 103 cycles is similar, but this does not show that the cyclable lithium has been consumed quantitatively. From the literature, we know that both CMC and SA, owing to their amorphous nature of their respective polymers, can form effective protective layers on the cathode materials even at lower contents (2 % or 3 %).^[35]^ This protective layer is robust and can effectively passivate high‐voltage spinel cathode materials like LNMO, thus resisting elevated‐temperature and high‐voltage degradation. This can effectively reduce the cyclable lithium intake and eventually decrease the internal resistance. However, the PVdF‐based binders can hardly cover and passivate cathodes uniformly and thus fails to alleviate the degradation of the electrolyte in contact with the active material.^[36]^

## Conclusion

LNMO electrodes with water‐soluble binders of SA and CMC compared to an LNMO electrode fabricated with a PVdF‐HFP binder provide similar electrochemical cycling performance at RT and at 55 °C. The discharge capacities and Coulombic efficiencies were however slightly better for the cells with SA. Also, the LNMO cathode with an SA binder showed the lowest internal resistance compared to LNMO with CMC; the internal resistance was the highest for LNMO with PVdF‐HFP both at RT and 55 °C. This lower internal resistance could be assigned to the protective effect of SA on the LNMO surface, or could be due its role in adsorbing the side products, which affects the intake of cyclable lithium. The overpotential compared to the LNMO cells with CMC and PVdF‐HFP was lower for the LNMO cell with SA, which is in line with the ICI results. XPS characterization of surface layers formed on LMNO and LTO electrodes and NMR‐spectrosopical measurements of liquid electrolytes showed similar results for all three cells, though the loss of cyclable lithium or the decomposition products in the electrolyte could not be quantified. This is in line with the observation that the color of the separator was changed after cycling, indicating the deposition of the side products, which again was not quantifiable. The HF concentration of the cycled electrolytes was much higher than that in the pristine electrolyte; however, the amount of HF was slightly lower in the cells with SA compared to the other cells. The TM dissolution showed similar results for all three cells, indicating that TM dissolution does not play a significant role in resistance increase here. Apart from all these factors affecting resistance increase, there could be other factors like surface structural reconstruction which is generally found in spinel oxide cathodes.[^37^, ^38^, ^39^] Hence, a question arising in this aspect would be if sodium alginate binders could help in decreasing the resistance during structural reconstruction of disordered phases of LNMO. Further investigations in this direction are required to get a better understanding of the role of sodium alginate.

## Experimental Section

LNMO powder was obtained from Haldor Topsøe A/S, Denmark. CMC was obtained from Leclanché, SA from Sigma‐Aldrich and PVdF‐HFP (Kynar Flex 2801) from Arkema. The LNMO composite electrodes were fabricated by mixing a slurry containing the active material powder with conductive carbon and binder at a weight ratio of 90 : 5 : 5 using a MM mixer mill (Retsch) at 25 Hz for 30 min. For CMC and SA binders, de‐ionized water was used as the solvent for blending the electrode mixture, while *N*‐methyl‐2‐pyrrolidone (NMP, VWR Chemicals) was used as the solvent for the PVdF‐HFP binder. The obtained slurries with different binders were then casted on carbon‐coated Al foil (20 μm thick) using a doctor blade. The coatings were then dried at 75 °C overnight to evaporate their respective solvents. Electrodes of diameter 20 mm were punched from each of the coatings and were calendared at a pressure of 1.59 ton cm^−2^. The calendared LNMO composite electrodes were then dried in a Buchi oven at 120 °C for at least 12 h. The active mass loading was around ≈10.5 mg cm^−2^ (or 1.5 mA h cm^−2^). The LNMO electrodes with different binders are designated as follows: LNMO_CMC for LNMO electrode with CMC binder, LNMO_SA for LNMO electrode with SA binder and LNMO_PVdF‐HFP for LNMO electrode with PVdF‐HFP binder. Commercial LTO electrodes coated with CMC‐SBR binder were provided by Leclanché which had a capacity loading of approximately 1.7 mA h cm^−2^. Electrochemical characterizations of the LNMO electrodes with different binders were evaluated in pouch cells. 1 m LiPF_6_ dissolved in a 1 : 1 mixture by volume of ethylene carbonate (EC) and diethyl carbonate (DEC) (Solvionic) was used as the electrolyte. The pouch cells were assembled in a glove box under argon atmosphere (H_2_O and O_2_ <1 ppm). The electrochemical instrument (BT‐2000, Arbin, USA) was employed to test the LNMO‐LTO pouch cells in a voltage range of 1.5–3.5 V. The internal resistance was measured using the intermittent current interruption (ICI) method consisting of 1 s rest at 2 min intervals as reported in Ref. [40]. Both the galvanostatic charge‐discharge cycling and the ICI measurements were performed at RT and 55 °C. Electrode morphologies were imaged via a Zeiss 1550 scanning electron microscope (SEM). Cycled pouch cells were opened in an Ar‐filled glovebox. LNMO and LTO electrodes were rinsed with DMC five times using 4–5 droplets each time to remove salt residues. Electrodes were placed on carbon tapes after drying off the DMC. Samples were transferred to the SEM in airtight glass vials and exposed to air for 15–20 s before being transferred to the SEM chamber. The accelerating voltage was 5 kV, and the working distance was 6 mm during analysis. Surface characterization was made via X‐ray photoelectron spectroscopy (XPS). The sample preparation was the same as for SEM and the airtight transfer system was used for sample to avoid any exposure to air. Pristine and cycled LNMO and LTO electrodes were analyzed using a Phi‐5500 instrument with monochromatized Al K_α_ radiation (1486.6 eV). Data calibration was made by linear shifting of the hydrocarbon peak to 285 eV for the LNMO data. CasaXPS was used for the analysis of XPS data. Inductively coupled plasma–optical emission spectroscopy (ICP‐OES) measurements were performed on the electrolyte retrieved from LNMO full‐cell with different binders. From each cell, 100 μL of the extracted electrolyte were transferred to 15 mL falcon tubes (VWR) for ICP‐OES analysis. Avio 500 Scott/Cross‐Flow Configuration was used for ICP‐OES measurements. The electrolyte was diluted by a factor of 100 with type 1 milliQ water (Fisher Scientific) containing 5 % HNO_3_ (Nitric acid 65 %, VWR) and filtered with 0.2 μm syringe filters (Whatman) before measurement. A calibration curve was formed for the measurements using a Multi‐element Calibration Standard (Mettler Toledo). Concentrations of 0, 0.05, 0.1, 1 and 10 ppm of the elements Li, Mn and Ni where used to create a 5‐point linear regression. All presented values are within an error of standard deviation of 5 %. HF content in the electrolyte retrieved from the cells was measured using a fluoride ion‐selective electrode (ISE, Mettler Toledo perfectION combination fluoride electrode). The procedure for the HF determination has been described by Strmcnik et al. and Tesfamhret et al.[^41^, ^42^] Solution NMR experiments were done with the samples retrieved from the electrolyte following the aforementioned process done in ambient air. The sample (50 μL) was diluted with anhydrous DMSO‐d_6_ solvent (400 μL) stored in ampoules and adding the soluble part to a glass NMR tube. ^1^H, ^19^F and ^31^P NMR spectra were recorded on a 400 MHz JEOL ECZ spectrometer. ^1^H spectra were internally referenced to DMSO‐d_6_ at 2.5 ppm (δ ^1^H), ^19^F spectra were internally referenced to BF_4_^−^ at −148 ppm (δ ^19^F) as it is the only common peak for all electrolytes, ^31^P spectra were internally referenced to PO_4_^3−^ at 0 ppm (δ ^31^P).^[34]^

1

## Supporting information

As a service to our authors and readers, this journal provides supporting information supplied by the authors. Such materials are peer reviewed and may be re‐organized for online delivery, but are not copy‐edited or typeset. Technical support issues arising from supporting information (other than missing files) should be addressed to the authors.

Supporting Information

## Data Availability

The data that support the findings of this study are available from the corresponding author upon reasonable request.
