# Supplementary material for: Investigation of Water‐Soluble Binders for LiNi0.5Mn1.5O4‐Based Full Cells
Source: ChemistryOpen. 2022 Jun 14;11(6):e202200065. doi: 10.1002/open.202200065 (PMC9197771; doi:10.1002/open.202200065)
Supplement: Supplementary file 1 — Supporting Information [file OPEN-11-e202200065-s001.pdf]

ChemistryOpen

Supporting Information

Investigation of Water-Soluble Binders for $LiNi_{0.5}Mn_{1.5}O_4$-Based Full Cells

Girish D. Salian, Jonathan Højberg, Christian Fink Elkjær, Yonas Tesfamhret, Guiomar Hernández, Matthew J. Lacey, and Reza Younesi*

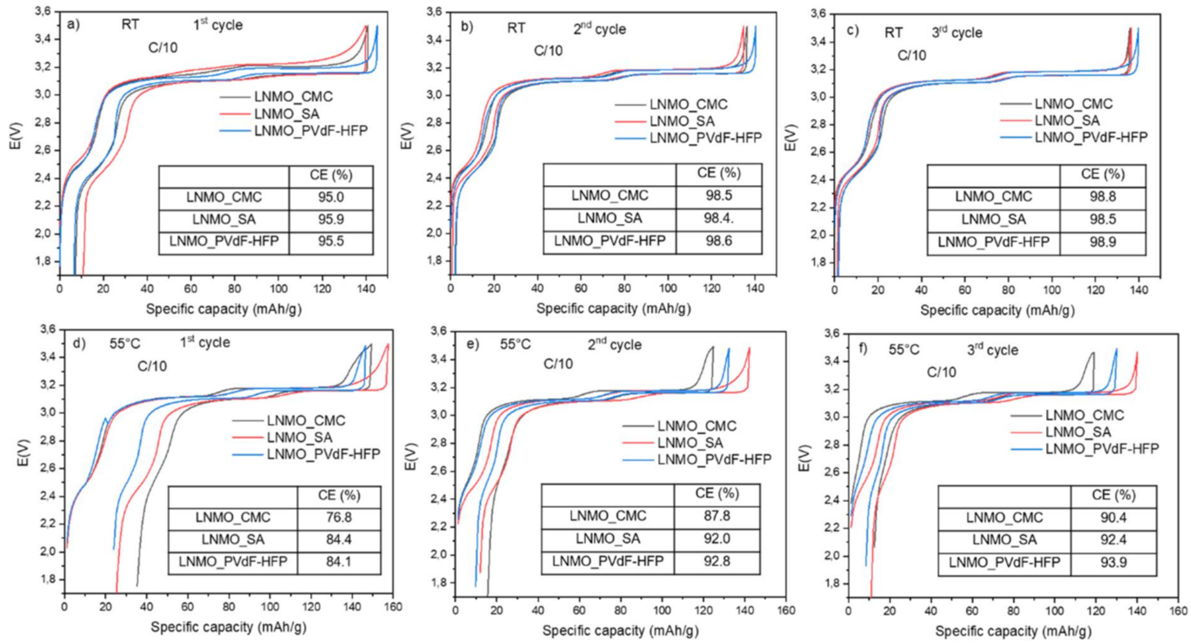

Figure S1. Galvanostatic voltage profiles of the formation cycles of LNMO_CMC, LNMO_SA and LNMO_PVdF-HFP full-cells at C/10 rate for the first three cycles a), b), c) at RT and d), e) and f) at 55 °C. Respective CE are given in the inset table.

The 1st formation cycles for all three cells at RT showed slight overpotential increase suggesting the formation of the passivation layer and this increase is relatively higher in the cells with LNMO_SA and LNMO_CMC. The 2nd and the 3rd cycles showed no difference in the overpotentials. The CE for the 1st cycles was around 95% which increases in 2nd and the 3rd cycles. For the cells cycled at 55 °C, all three cycles show very low overpotentials compared to the cycles at RT due to higher kinetics at 55 °C. The 1st cycle CE for LNMO_CMC is around 76%, which suggests higher lithium consumption during the 1st cycle. The CE increases with subsequent cycles for all the cells.

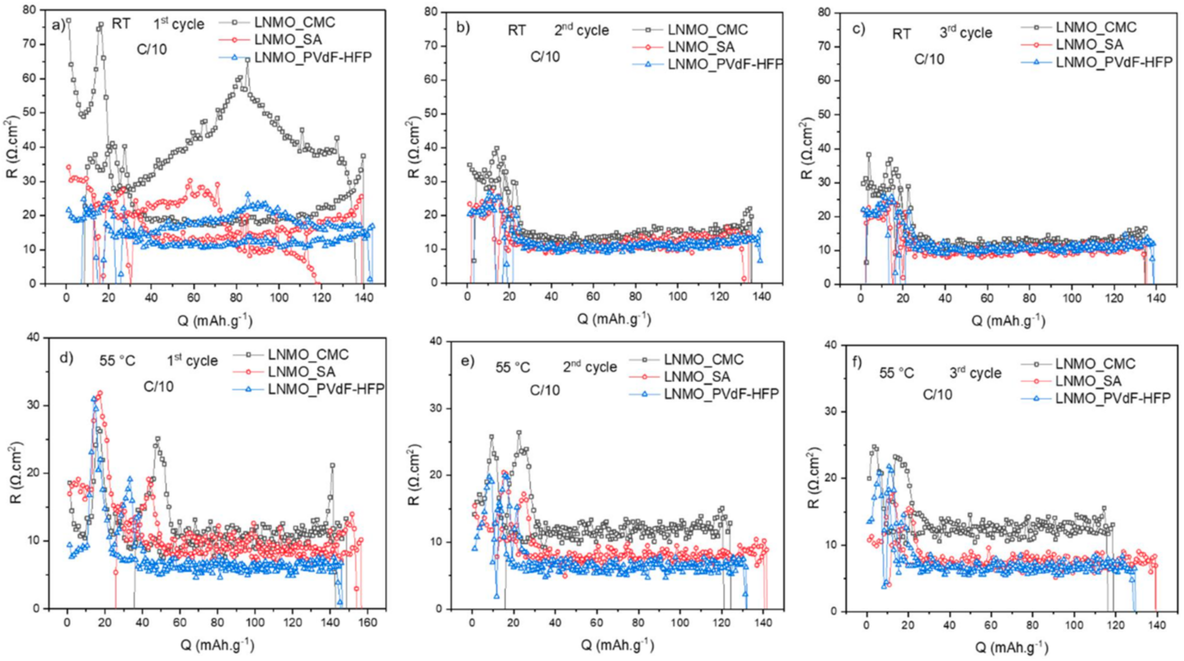

Figure S2. Resistance profiles of the ICI measurements of LNMO-LTO full-cells for the formation cycles at C/10 with the three different binders performed at RT (a, b, c), and at 55 °C (d, e, f).

The ICI measurements for the 1st cycles at RT for all the three cells show a resistance increase for LNMO_CMC. This could come from the surface layer formation which is relatively thicker than that for LNMO_SA and LNMO_PVdF-HFP. The 2nd and the 3rd cycles do not show marked differences in resistances. For the ICI measurements of the cells tested at 55 °C, the resistances for LNMO_CMC increases slightly up to the 3rd cycles compared to the other two binders. But interestingly the resistances at 55 °C for all the three binders are similar to that at RT. As mentioned earlier, the faster/improved kinetics at elevated temperatures is the reason.

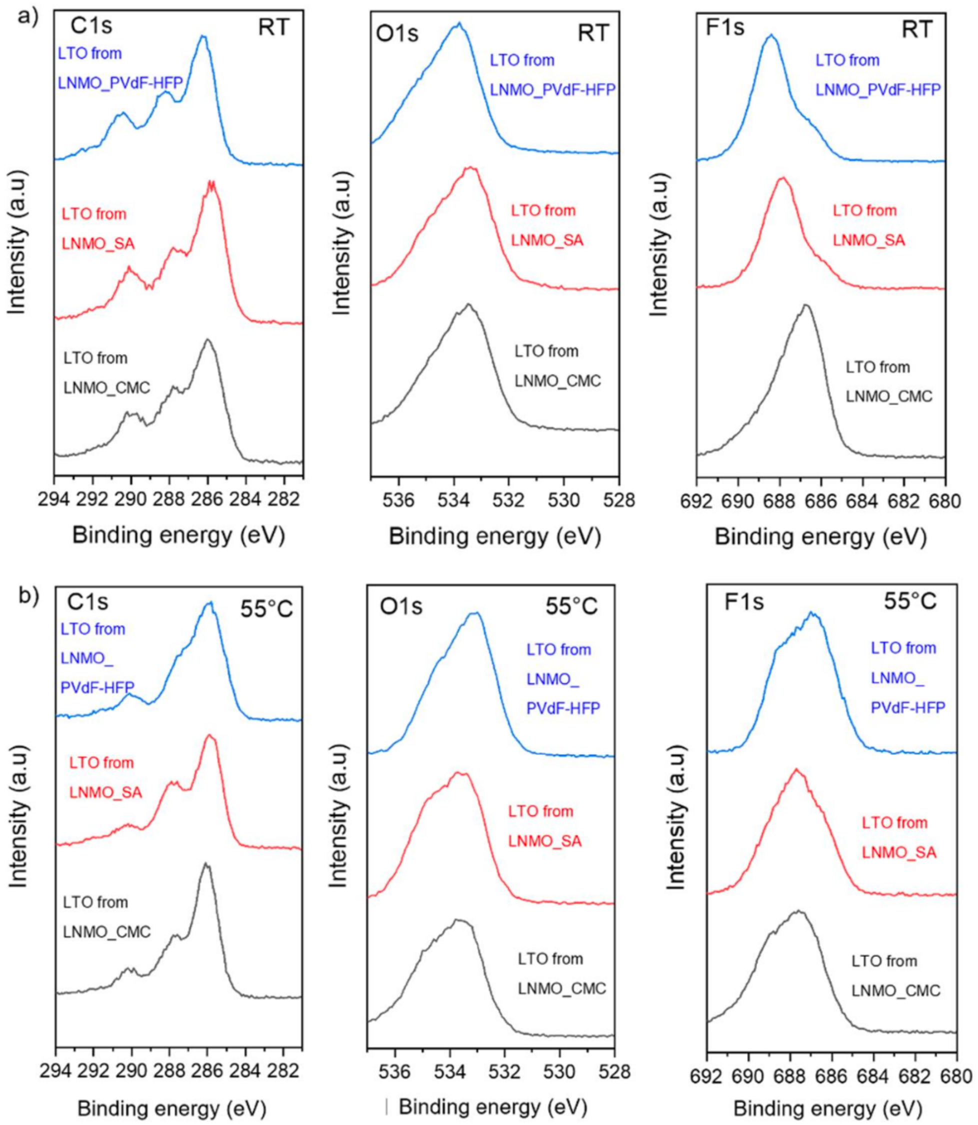

Figure S3. XPS spectra of the LTO electrodes from their respective full cells a) at RT, b) at 55 °C.

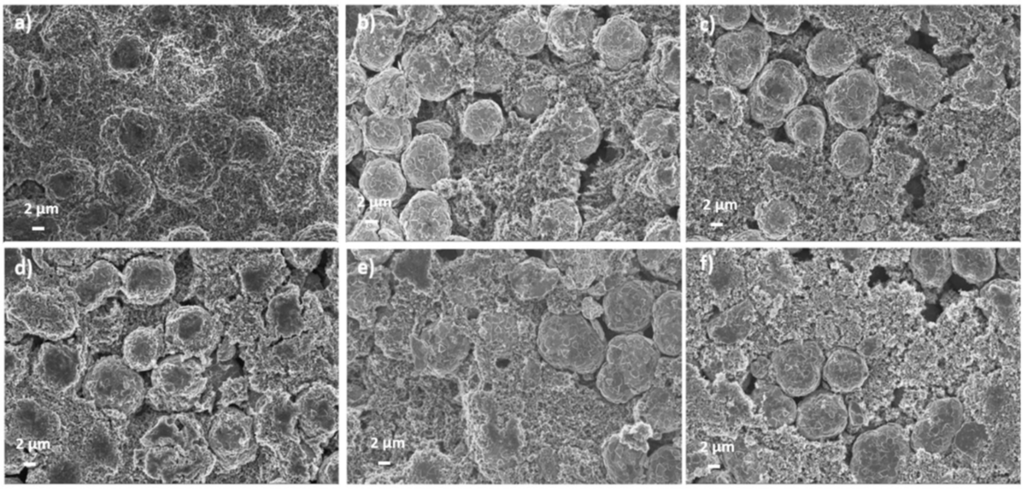

Figure S4 SEM images of the LNMO electrodes after 100 cycles at RT (a, b, c) and at 55 °C (d, e, f) for LNMO_CMC, LNMO_SA and LNMO_PVdF-HFP, respectively.

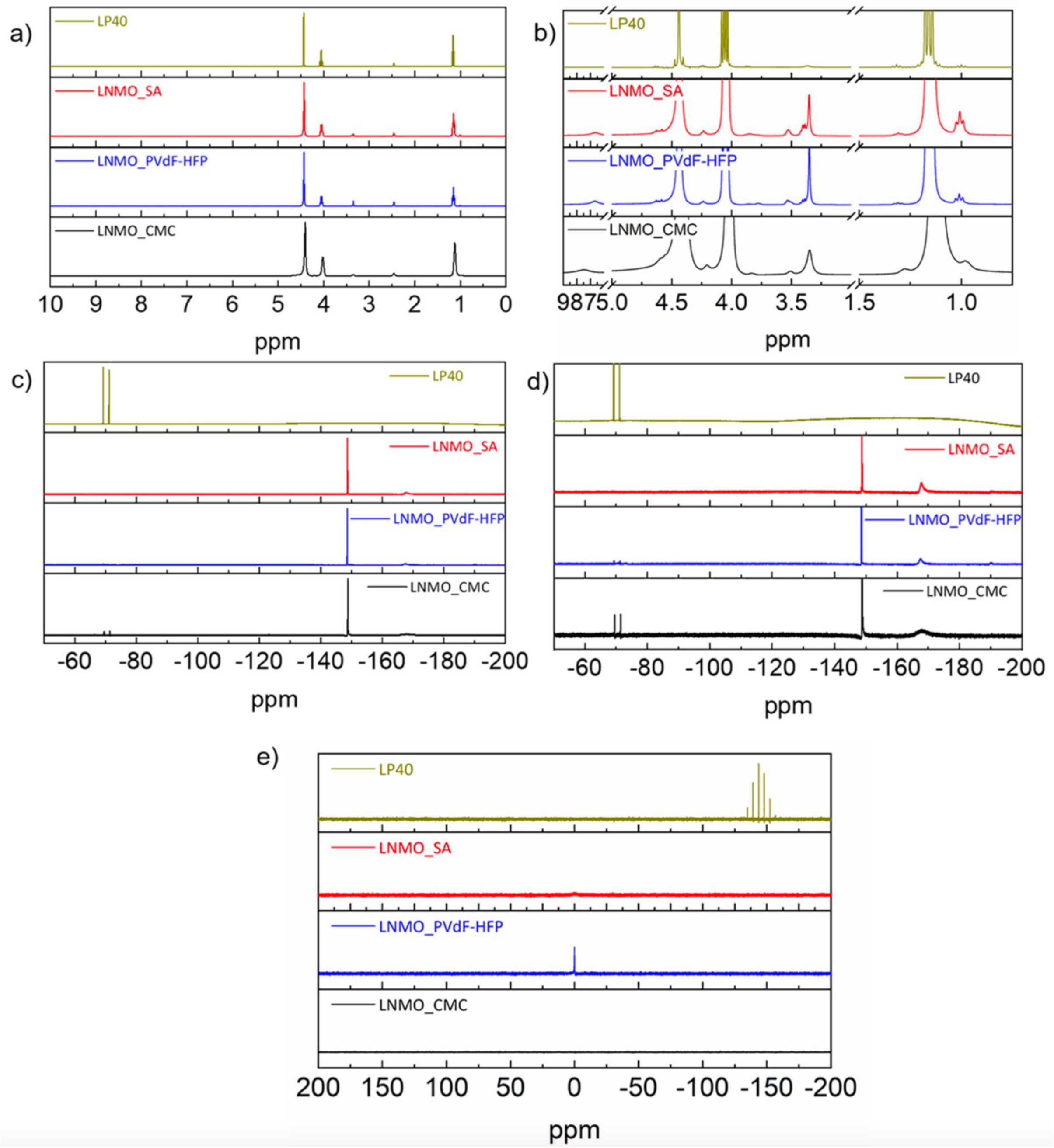

Figure S5. ^{1}H NMR spectra (a) full scale and (b) enlarged; ^{19}F NMR spectra (c) full scale and (d) enlarged; e) ^{31}P NMR spectra of the electrolyte retrieved from the cells cycled at 55 °C.

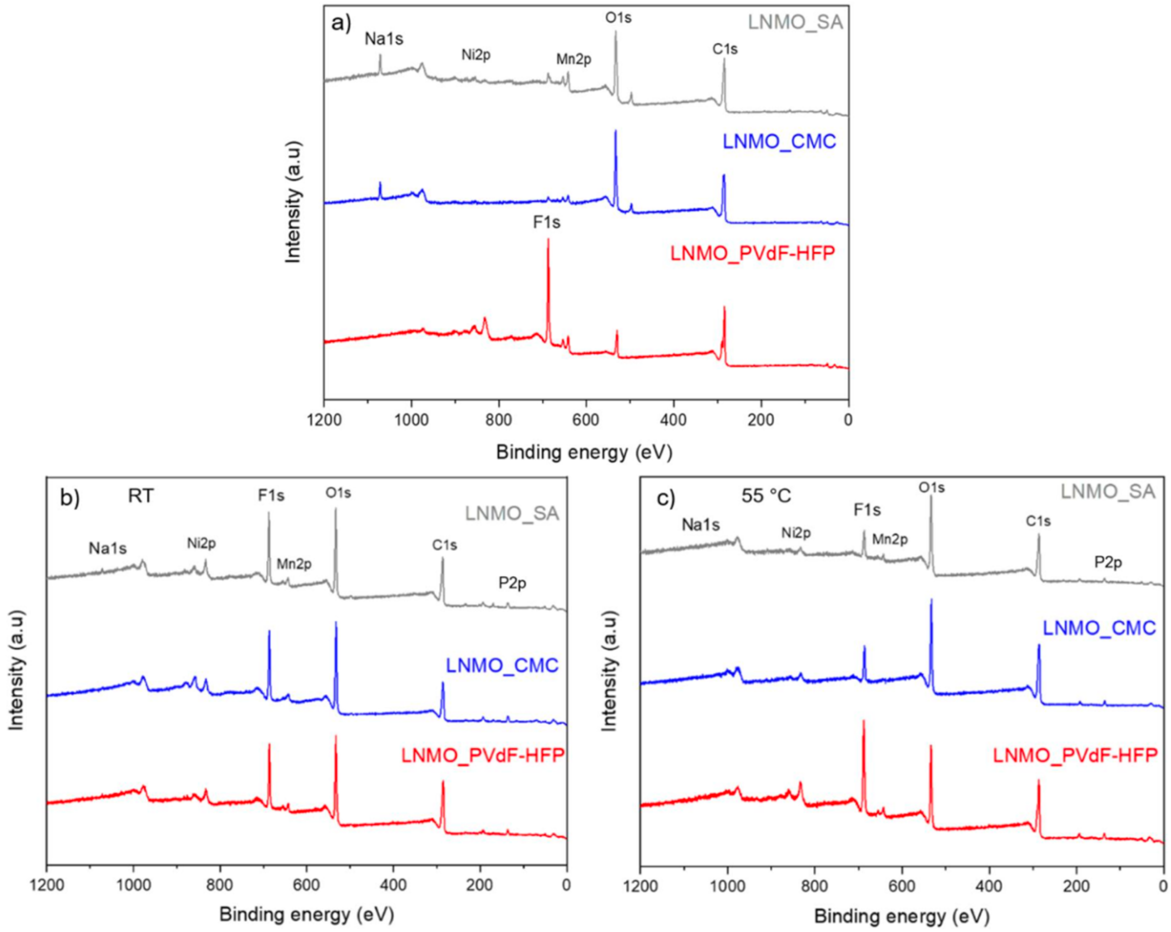

Figure S6. XPS survey spectra of a) pristine LMNO electrode, and LNMO electrodes cycled for 100 cycles at b) RT and c) 55 °C.
